# Measurement of a model of implementation for health care: toward a testable theory

**DOI:** 10.1186/1748-5908-7-59

**Published:** 2012-07-03

**Authors:** Joan M Cook, Casey O’Donnell, Stephanie Dinnen, James C Coyne, Josef I Ruzek, Paula P Schnurr

**Affiliations:** 1 Department of Psychiatry, Yale School of Medicine, 950 Campbell Avenue, NEPEC/182, West Haven, CT, 06516, USA; 2 National Center for PTSD, 215 North Main Street, White River Junction, VT, 05009, USA; 3 Department of Psychiatry, Perelman School of Medicine of the University of Pennsylvania, 423 Guardian Drive, Philadelphia, PA, 19104, USA; 4 Department of Psychology, University of Groningen, the Netherlands, P.O. Box 660, 9700 AR, Groningen, The Netherlands; 5 Department of Psychiatry, Stanford University-Menlo Park Division, 795 Willow Road, Menlo Park, CA, 94025, USA; 6 Department of Psychiatry, Geisel School of Medicine, Dartmouth College, 1 Rope Ferry Road, Hanover, NH, 03755, USA

## Abstract

**Background:**

Greenhalgh et al. used a considerable evidence-base to develop a comprehensive model of implementation of innovations in healthcare organizations [1]. However, these authors did not fully operationalize their model, making it difficult to test formally. The present paper represents a first step in operationalizing Greenhalgh et al.’s model by providing background, rationale, working definitions, and measurement of key constructs.

**Methods:**

A systematic review of the literature was conducted for key words representing 53 separate sub-constructs from six of the model’s broad constructs. Using an iterative process, we reviewed existing measures and utilized or adapted items. Where no one measure was deemed appropriate, we developed other items to measure the constructs through consensus.

**Results:**

The review and iterative process of team consensus identified three types of data that can been used to operationalize the constructs in the model: survey items, interview questions, and administrative data. Specific examples of each of these are reported.

**Conclusion:**

Despite limitations, the mixed-methods approach to measurement using the survey, interview measure, and administrative data can facilitate research on implementation by providing investigators with a measurement tool that captures most of the constructs identified by the Greenhalgh model. These measures are currently being used to collect data concerning the implementation of two evidence-based psychotherapies disseminated nationally within Department of Veterans Affairs. Testing of psychometric properties and subsequent refinement should enhance the utility of the measures.

## Background

There is currently a wide gap between what treatments have been found to be efficacious in randomized controlled trials and what treatments are available in routine clinical care. One comprehensive theoretical model of dissemination and implementation of healthcare innovations intended to bridge this gap was developed by Greenhalgh et al. [1]. Derived from a systematic review of 13 distinct research traditions [2,3], this model is both internally coherent and based largely on scientific evidence. The model is consistent with findings from other systematic narrative reviews [4-6] regarding the factors found to be related to implementation. In addition, it served as the starting point for development of the Consolidated Framework for Implementation Research [7].

**Figure 1:**
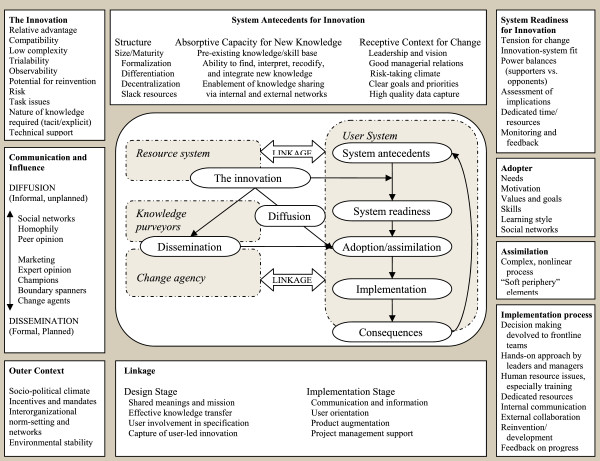
Greenhalgh and colleagues (2004) model of Implementation processes.

As shown in Figure 1, implementation is viewed as complex processes organized under six broad constructs: innovation; adopter; communication and influence; system antecedents and readiness (inner organizational context); outer (inter-organizational) context; and implementation process. However there are no explicit recommendations for operational definitions or items to measure most of the identified constructs. The authors recommend a structured, two-phase approach for capturing their model [1]. For phase one, they advised assessment of specific individual components of the model (i.e., perceived characteristics of the innovation, adopter characteristics). For the second phase, they proposed construction of a broad, unifying meta-narrative of how these components interact within the social, political, and organizational context [8].

In order to advance toward a testable theory and thus benefit implementation science, an operationalization of key constructs and their measurement is needed. Articulation of this model may also aid the implementation process in other ways. For example, administrator or treatment developers may ask providers to complete these measures in order to understand individual and organizational barriers to implementation and to identify strengths that can help teams overcome these challenges. This information can then be used to inform design of training, help promote provider engagement in evidence-based innovations, assist in problem-solving with obstacles, and guide development of the implementation process.

Our research group set out to operationalize the constructs in Greenhalgh et al.’s [1] model for use in a quantitative survey and a semi-structured interview guide (a full copy of the survey can be found in Additional file 1 and a full copy of the semi-structured interview in Additional file 2). The present paper provides the background, rationale, working definitions, and measurement of constructs. This work was done in preparation to study a national roll-out of two evidence-based psychotherapies for post-traumatic stress disorder (PTSD) within the Department of Veterans Affairs (VA) [9]. Although the questionnaire and interview guide were developed to assess factors influencing implementation of specific treatments for PTSD, they can likely be adapted for assessing the implementation of other innovations. This systematic effort represents a first step at operationalizing constructs in the Greenhalgh model.

## Methods

### Construction of measures: systematic literature search and article review selection process

Measure development began with a systematic literature search of keywords representing 53 separate sub-constructs from the six broad constructs (innovation, adopter, communication and influence, system antecedents and readiness, outer context, and implementation process) identified in Figure 1. Only those constructs that were both related to implementation of an existing innovation (rather than development of an innovation) and were definable by our research group were included.^1^ Searches were conducted in two databases (PsycInfo and Medline) and were limited to empirical articles published between 1 January 1970 and 31 December 2010. Search terms included the 53 sub-constructs (e.g., relative advantage) and ‘measurement’ or ‘assessment’ or ‘implementation’ or ‘adoption’ or ‘adopter’ or ‘organization.’ After culling redundant articles, eliminating unpublished dissertations and articles not published in English, we reviewed abstracts of 6,000 remaining articles. From that pool, 3,555 citations were deemed appropriate for further review.

Two members (CO, SD) of the investigative team conducted a preliminary review of titles and abstracts for possible inclusion. Articles were selected for further consideration if they proposed or discussed how to measure a key construct. Clear and explicit definitions of constructs were rarely provided in the literature, resulting in our inclusion of articles with concepts that overlapped with one another. From the review of titles and abstracts, 270 articles were retrieved for full text review. If the actual items from the measure were not provided in the paper, a further search was made using cited references. The investigative team also reviewed surveys that had been used in studies on health providers’ adoption of treatments [10-12] and organizational surveys related to implementation [13,14].

We next developed a quantitative survey and semi-structured interview using an iterative process whereby the full investigative team reviewed potential items. In order for inclusion of an item in our measurement approach, all members of the team had to agree. The resulting pool of items was presented to 12 mental health professionals who offered feedback on item redundancy and response burden. Items were further revised by the team for clarity and consistency. In addition, our team concluded that it would be burdensome to participants if we included items reflecting every aspect of the model in the quantitative survey. Therefore, we made strategic decisions, described below, as to which items to retain in the survey versus the semi-structured interview. Certain constructs in the Greenhalgh model appear under more than one domain (e.g., social network appears under both adopter and communication and influence) or assess overlapping constructs (e.g., peer and opinion leaders). For certain constructs the use of administrative data was deemed as the most efficient means of assessment and served to augment survey or interview questions (e.g., incentives and mandates, environmental stability).

## Results

Table 1 presents the constructs and working definitions as well as a sample item for each. For each construct, an overview of relevant measures is provided followed by explanation of the measures that ultimately influenced our survey and semi-structured interview questionnaires, or for relevant constructs the use of administrative data.

**Table 1:** Model constructs and examples of survey and interview questions and administrative data

**Construct**	**Operational definition**	**Example of survey question**	**Example of interview question**	**Example of administrative data**
**Innovation**				
Relative advantage	Degree to which the innovation is considered superior to existing practices.	[The treatment] is more effective than the other therapies I have used.	N/A	N/A
Compatibility	Innovations’ consistency with existing values, experiences, and needs of adopter and system.	Using [the treatment] fits well with the way I like to work.	N/A	N/A
Complexity	Level of difficulty to understand and use the innovation.	[The treatment] is easy to use.	N/A	N/A
Trialability	Ability to experiment with the innovation on a limited or trial basis.	It is easy to try out [the treatment] and see how it performs.	N/A	N/A
Observability	Innovations’ results are observable to others.	[The treatment] produces improvements in my patients that I can actually see.	N/A	N/A
Potential for reinvention	Ability to refine, elaborate and modify the innovation.	[The treatment] can be adapted to fit my treatment setting.	N/A	N/A
Risk	Risk or uncertainty of outcome associated with the innovation.	Using [the treatment] includes a risk of worsening patients’ symptoms.	N/A	N/A
Task issues	Concerns about the innovation that need to be focused on to accomplish implementation.	Using [the treatment] improves the quality of work that I do.	How effective is [the treatment] when presenting problems are more acute, severe or complicated?	N/A
Nature of knowledge	Information about the innovation can be codified and transferred from one context to another.	The knowledge required to learn [the treatment] can be effectively taught.	N/A	N/A
Technical support	Available support components (e.g., training, manuals, consultation help desk).	There is adequate consultation to support me in implementing [the treatment] in my setting.	What are some of the supports or structures that are helpful in implementing [the treatment]?	N/A
**Adopter Characteristics**				
Needs	Observed or experienced deficit in an adopter’s practice or organizational setting.	I feel the need to learn additional therapies to help my patients with their symptoms.	N/A	N/A
Motivation	Adopter’s interest and willingness to learn new things.	I am actively working on improving my therapy techniques.	What was your interest in attendance and involvement with training in [the treatment]?	N/A
Values and goals	What adopters place value in and what are their intended goals for treatment.	I think it is important that providers use evidence-based treatments.	N/A	N/A
Skills	Adopter’s context specific skill set.	Level of training in evidence-based treatment.	Have you been trained in [the treatment]?; How far along in the training process did you go?	N/A
Learning style	Adopter’s consistent patterns in perceiving, remembering, judging and thinking about new information.	I learn effectively through experience, such as role-play or work with actual patients.	What is your preferred way of learning a new approach to treatment?	N/A
Locus of control	Adopter’s belief that events are under one’s personal control (internal) or that events are largely a matter of chance or due to external events (external).	My life is determined by my own actions.	N/A	N/A
Tolerance of ambiguity	Adopter’s ability to accept uncertainty.	I am comfortable not being able to predict how a new treatment will work for a particular patient.	N/A	N/A
Knowledge-seeking	Adopter’s autonomous efforts to attain knowledge/information.	I regularly try to improve my psychotherapy skills.	Can you describe the experience of learning [the treatment]? Were there elements that were more or less difficult to learn?	N/A
Tenure	Length of employment in setting and in field.	Number of years with program. Year provider received highest professional degree.	N/A	N/A
Cosmopolitan	Adopter’s strong connections with professional network; Engagement and attendance at professional meetings and other informational venues.	I attend national conferences related to my work with patients.	N/A	N/A
**Communication and Influence**				
Social networks	Structure and quality of social network, both formal and informal.	When you need information or advice about psychotherapies, to which other providers in your treatment setting do you usually turn?	N/A	N/A
Homophily	Degree of similarity (e.g., experiences, values, social status) among providers targeted for implementation.	N/A	*See pre-existing knowledge and skills	N/A
			Information compiled across each setting to assess for similarity among degree, discipline and theoretical orientation.	
Peer opinion leader	Internal member of the social network able to exert influence on providers’ beliefs and actions through representativeness and credibility (can be positive or negative).	I have at least one colleague in my treatment setting who I trust as a resource of information regarding [the treatment].	N/A	N/A
Marketing	Process of promoting, selling and distributing a treatment.	N/A	How were you persuaded [the treatment] would meet your clinical needs and those of your patients?	N/A
Expert opinion leader	Senior or high status formal authority with reputable expertise.	I am a consultant or trainer in an evidence-based psychotherapy.	Do you have access to an expert consultant?	N/A
Champions	Individuals who support and promote the innovation through its critical stages.	N/A	Were there key individuals in your program that rallied to support and promote [the treatment]?	N/A
Boundary spanner	An individual who is part of the work environment and part of the innovation technology (e.g., trainer in the innovation).	I have at least one readily accessible person who enables me to connect with experts.	N/A	N/A
Change agents	An individual who is a facilitator of change in various stages from problem identification or translation of intent into action.	N/A	Was there an individual(s) responsible for facilitating implementation of [the treatment]?	N/A
**System Antecedents for Innovation**				
**Structure**				
Size/Maturity	Number and experience of providers; Date of program inception.	N/A	N/A	Details of the program such as number of available beds, past-year patients served and number of full-time providers at various educational levels.
Formalization	Degree to which an organization is run by rules and procedures.	N/A	Do you feel that the rules are clear in your organization for making decisions and implementing changes?	National monitoring data concerning program adherence to patient admission, discharge and readmission procedures.
Differentiation	Complexity of the program in terms of structure, departments or hierarchy.	N/A	How do different levels of care communicate and share treatments? (e.g., outpatient and residential care)	N/A
Decentralization	Extent to which locus of authority and decision-making are dispersed throughout an organization.	N/A	How did the program make the decision to implement [the treatment] (or not)?	N/A
Slack resources	Actual versus spent budget and/or the total potential hours each provider is available versus actual time spent working.	N/A	N/A	Staff to patient ratio; Program capacity (number of unique patients, number of unique visits).
**Absorptive Capacity for Knowledge**				
Preexisting knowledge/skill base	Adopters’ level of preexisting knowledge and skills.	Adopters’ professional discipline and degree.	What is your professional background?	N/A
Ability to learn and integrate new information	Adopters’ capacity to take in new data and incorporate it with existing knowledge.	N/A	*See Knowledge-seeking	N/A
Enablement of knowledge sharing	Creation of venues for sharing information.	There are adequate communication systems to support information exchange in my treatment setting.	N/A	N/A
**Receptive Context for Change**				
Leadership and vision	Style of leadership and presence of identified and articulated trajectory with guided direction toward implementation.	Program leaders in my treatment setting are actively involved in supporting the evidence-based therapy initiatives.	To what extent is [the treatment] supported by program leaders and supervisors?	N/A
Managerial relations	Relationship between staff and program leadership.	Program leaders and staff in my treatment setting have good working relationships.	Do program leaders and staff work well together?	N/A
Risk-taking climate	A work environment that encourages experimentation with new practices, ideas and technologies.	My work environment encourages experimentation with new practices.	Does your work environment allow opportunities to experiment with new treatments?	N/A
Clear goals and priorities	Explicitness of organizational purposes and aims.	The goals and priorities of my treatment setting are clear and consistent.	N/A	Program mission statement or related document(s).
High quality data capture	Utilization of context specific data in implementation process.	Outcome data are routinely used in my treatment setting for quality improvement.	N/A	N/A
**System Readiness for Innovation**				
Tension for change	Perceived need for change to an organization’s current provision of services.	N/A	Did other providers in your setting see a need to make changes to the program and treatment approaches?	N/A
Innovation-system fit	Compatibility of the innovation with the organizational setting and structure.	N/A	To what extent does [the treatment] fit with the interventions offered in your treatment setting?	N/A
Power balances	Relative power of groups invested in implementation (e.g., program staff, director, management).	N/A	Was there agreement among providers, director and management regarding implementation?	N/A
Assessment of implications	Estimation of perceived benefits and consequences of implementation.	N/A	Have there been any unintended benefits or consequences to implementing [the treatment]?	N/A
Dedicated time and resources	Available means needed to implement an innovation (e.g., funding, time, access, administrative support, etc.).	There is adequate time to implement [the treatment] in my treatment setting.	Was there sufficient time and resources available to implement [the treatment]?	N/A
Monitoring feedback	Providers’ formal and informal opinions on efforts to implement.	N/A	Were there opportunities for you to provide and receive feedback about the implementation process?	N/A
**Outer Context**				
Socio-political climate	Social and political factors within the organization affecting implementation.	N/A	Did you feel pressure to adopt [the treatment]?	N/A
Incentives and mandates	Implicit or explicit inducements, encouragements, or directives to implement.	I am expected to use [the treatment] as part of my job.	N/A	National mandates in provider handbooks.
Inter-organizational norm-setting and networks	Implicit or explicit rules defining acceptable behavior; How information is exchanged within the larger organization.	N/A	What is your understanding of expectations in regards to [the treatment] implementation and the associated rewards and penalties?	N/A
Environmental stability	Status of funding and persistence of goals.	N/A	What staffing or funding changes have occurred in the recent past?	N/A
**Implementation Process**				
Decision-making	Evaluative process in selecting a treatment from available options.	N/A	*See Decentralization	N/A
Hands-on approach by leaders	Direct involvement and oversight of procedure and policy.	Program leaders in my treatment setting are actively involved in daily program activities.	N/A	N/A
Human resources issues	Adequacy of education and training at all levels of the program workforce.	N/A	N/A	Information on staff degree status and clinical training; Clinical position vacancies.
Internal communication	Process by which information is exchanged between individuals within the program.	N/A	Did you seek consultation from someone in your setting regarding [the treatment] or its implementation process?	N/A
External communication	Process by which information is exchanged between providers within the program and outside stakeholders.	N/A	Did you seek consultation from someone outside your setting regarding [the treatment] or its implementation process?	N/A
Reinvention	Extent to which the innovation can be changed in the process of implementation.	N/A	How do you (or your program) use [the treatment)? Do you use the full protocol (exact number of sessions, in order, including all content), or have the protocols required modification?	N/A
Feedback	Information exchange between program staff and external stakeholders.	N/A	*See Monitoring feedback	N/A

### Innovation

The five innovation attributes originally identified by Rogers [2] and included in the Greenhalgh et al. model are: relative advantage, compatibility, complexity, trialability, and observability. Additional perceived characteristics given less emphasis by Rogers but included by Greenhalgh et al. are potential for reinvention, risk, task issues, nature of the knowledge required for use, and augmentation/technical support.

Several investigators have attempted to operationalize Rogers’ innovation attributes [14-18]. The approach most theoretically consistent with Rogers was constructed by Moore and Benbasat [19], but this was not developed for application to a healthcare innovation [20,21]. The 34- and 25-item versions of that scale have high content and construct validity and acceptable levels of reliability. Our group used several items from the Moore-Benbasat instrument that were deemed applicable to mental health practice (i.e., complexity, observability, trialability, compatibility) and reworded others to be more relevant to healthcare treatments (e.g., ‘The treatment [name] is more effective than the other therapies I have used’).

Others have also assessed Rogers’ innovation characteristics. A questionnaire by Steckler et al. [17] further informed the phrasing of our survey items for content and face validity. Content from additional sources [14,18,22,23] was deemed not applicable because it examined socio-technical factors, deviated too far from the constructs, or did not map onto measurement of a healthcare practice.

Items concerning potential for reinvention were not taken from existing surveys as most focused on identifying procedures specific to a particular intervention [24]. Thus, we were influenced by other discussions of reinvention as they more broadly applied across implementation efforts [25]. In particular, our items were constructed to assess providers’ reasons for making adaptations. As a perceived attribute of innovation, risk refers to uncertainty about the possible detrimental effects. Existing tools for assessing risk focus on the adopter rather than the innovation [26,27]. Thus, we reviewed these instruments for the adopter characteristics (presented below) as well as utilizing them to inform our items for risk.

The limited literature on nature of knowledge involves how information is instrumentally used both for problem solving and strategic application by adopters [28]. However, Greenhalgh viewed nature of knowledge as whether an innovation was transferable or codifiable. This required us to craft our own items. Assessment of technical support is typically innovation specific, such as adequate support for a technology or practice guideline [29,30]. Technical support needed to acquire proficiency is likely different across innovations (i.e., training support), and thus we included items on the helpfulness of manuals and accompanying materials. Davis [31] developed a reliable and valid instrument to assess perceived usefulness (i.e., belief that the innovation enhances job performance). Although the construct has a different label, we judged it as nearly identical to Greenhalgh’s task issues. One item was borrowed from this scale to represent task issues.

All innovation attributes in the Greenhalgh model were represented in the quantitative survey. A couple (e.g., technical support) were also included in the semi-structured interview.

### Adopter characteristics

Greenhalgh et al. [8] suggested that a range of adopters’ psychological processes and personality traits might influence implementation. Items specifically identified in the model include adopter needs, motivation, values and goals, skills, learning style, and social networks [8]. Not all proposed adopter characteristics were depicted in the model figure; Greenhalgh [1] identified other potentially relevant adopter characteristics such as locus of control, tolerance of ambiguity, knowledge-seeking, tenure, and cosmopolitan in the text.

There was a lack of operational definitions in the literature regarding need; thus, we created our own. Assessment of this construct was informed by questions from the Texas Christian University Organizational Readiness to Change survey [12]. We included one item in our survey specific to need in the context of professional practice.

Assessing motivation or desired levels and ‘readiness for change’ has most often been based on the transtheoretical stages of change model [32-34]. One of the most widely used tools in this area is the University of Rhode Island Change Assessment Scale [33], which has items assessing pre-contemplation (not seeking change), contemplation (awareness of need for change and assessing how change might take place), action (seeking support and engaging in change), and maintenance (seeking resources to maintain changes made). We adapted items from this scale for our survey. Continued development of the stages of change model after construction of the Change Assessment Scale incorporated an additional preparation stage, which we represented in the qualitative interview as a question regarding providers’ interest in and attendance at trainings in evidence-based treatments.

Assessment of values and goals typically reflect estimation of personal traits/values (e.g., altruism) and terminal goals (e.g., inner peace) [34]. Funk et al. [35] devised a survey that included some adopter characteristics in relation to utilizing research-based innovations in healthcare settings. We used an item from their survey [35] as well as one from the Organizational Readiness to Change-Staff Version survey [12] to operationalize this construct.

The preferred means of assessing skills in healthcare practice is observational assessment as opposed to self-report [36,37]. However, in order to capture some indication of skill, we simply added an ordinal item on level of training in the evidence-based treatment.

Greenhalgh et al. [1] provided no formal definition of learning style. We reviewed numerous learning style measures [38-45], but most had poor reliability and validity [46]. Others had attempted to revise and improve upon these instruments with limited success [47,48]. Recently, an extensive survey of learning style was created [49]. Although we did not utilize these items due to their lack of reflection of learning processes (e.g., auditory), we did follow the suggestion to directly word items about preference of instructional methods [49] (for reviews see [50,51]). Due to the potential complexity of this construct and the various ways to measure it, we included three diverse items not expecting them to necessarily represent one scale and also assessed this in the interview.

Measurement of some of the adopter traits has occurred in the larger context of personality research. For example, there are several measures of locus of control [52-54]. After a review of these tools and discussion as to what was most applicable to the implementation of healthcare innovations, our group primarily borrowed items from Levenson’s [53] Multidimensional Locus of Control Inventory. The Levenson inventory includes three statistically independent scales that allow a multidimensional conceptualization of locus of control unlike the widely used Rotter scale, which is unidimensional and conceptualizes locus of control as either internal or external. The Levenson scale has strong psychometric properties [53]. Unlike other LOC scales, it is not phrased to focus on health and therefore appeared more easily applied to measure LOC as a general personality factor. Similarly, numerous surveys of tolerance (and intolerance) for ambiguity have been developed [55-61]. After reviewing these measures, we chose to adapt items from McLain’s [59] Multiple Stimulus Types Ambiguity Scale due to its relevance to healthcare.

For knowledge-seeking, we adapted one additional question from the Organizational Readiness to Change-Staff Version survey [12] and devised two of our own. Tenure has consistently been measured as a temporal variable [62-64]. A clear distinction can be made between organizational and professional tenure. For the purposes of our survey, both organizational tenure [64] and professional tenure were included.

One means of assessing cosmopolitanism is by identifying belonging to relevant groups [65]. Woodward and Skrbis’ [66] assessment of cosmopolitanism informed the construction of our items. Pilcher [65] differentiated between two conceptualizations of cosmopolitanism: ‘subjective/identity’ and ‘objective/orientation,’ where the former captures affiliations and the latter relevant attitudes. We followed a more ‘subjective/identity’ approach by including one survey item, capturing how many professional meetings one attends per year [67].

### Communication and influence

Communication and influence constructs in the Greenhalgh model included in the survey are: social networks, homophily, peer opinion (leader), marketing, expert opinion (leader), champions, boundary spanners, and change agent.

One of the most common measures of social networks is a name generator response used to map interpersonal connections [68-70]. Relatedly although there are several ways that peer opinion leaders have been assessed [3,71], the most common is to ask respondents from whom they seek information and advice on a given topic. We included a name generator in the survey to identify social networks as well as items asking about peer relationships. Similarly we asked one item to assess whether a provider had access to a peer opinion leader. This latter item is modeled after the Opinion Leadership scale, which has adequate reliability [72].

Since there was no psychometrically sound measure of homophily in the literature [73], we chose to capture this construct from the interview data in regards to the degree to which providers in a particular program had similar professional and educational backgrounds and theoretical orientations. Similarly, there was no identified measure of marketing, thus we crafted one question for the interview.

While the terms expert opinion leader, change agent and peer opinion leader are often used interchangeably and inconsistently [8], we were careful to create distinct definitions and measurements for each of these. In regards to measurement of an expert opinion leader, in the interview, we assessed access to an expert consultant, and in the survey, we ask if the provider themselves is a consultant or trainer in the treatment.

Innovation champions play multiple roles in promotion (e.g., organizational maverick, network facilitator [1,15,74]). Our team assessed this construct in the interview by initiating a discussion of how the innovation was promoted and by whom.

The construct of boundary spanners has received minimal application in studies of implementation in healthcare settings [75]. Because there were no available tools for this construct, we modeled our items from the definition of boundary spanners—individuals who link their organization/practice with internal or external influences, helping various groups exchange information [76]. We also utilized one question to capture the concept of whether providers were affiliated with or were themselves boundary spanners.

The interview also included questions to identify the influence of a change agent by asking about decision-making responsibility in the organization as well as facilitation of internal implementation processes. Thus, while only a limited number of constructs within the communication and influence section were included in the survey, many of the concepts seemed best captured through dialogue and description and thus were included in the interview.

### System antecedents and readiness for innovation (inner context)

The constructs that comprise the inner and outer organizational context overlap considerably, making sharp distinctions difficult [6,77]. Greenhalgh identified two constructs of inner context: system antecedents (i.e., conditions that make an organization more or less innovative) and system readiness (i.e., conditions that indicate preparedness and capacity for implementation).

As can be seen in Figure 1, system antecedents for innovation include several sub-constructs organizational structure (size/maturity, formalization, differentiation, decentralization, slack resources); absorptive capacity for new knowledge (pre-existing knowledge/skills base, ability to interpret and integrate new knowledge, enablement of knowledge sharing); and receptive context for change (leadership and vision, good managerial relations, risk-taking climate, clear goals and priorities, high-quality data capture). In a review of organizational measures related to implementation in non-healthcare sectors, Kimberly and Cook [14] noted few standardized instruments.

Measurement of organizational structure has typically used simple counts of particular variables. Although this appears straightforward, providers may be limited in their knowledge of their organizational structure [14]. Thus organizational structure and its sub-constructs deemed best captured through the interview and administrative data sources. For our investigation of national roll-outs of two evidence-based psychotherapies, we were also able to integrate existing data routinely collected by the VA’s Northeast Program Evaluation Center (NEPEC). NEPEC systematically collects program, provider, and patient level data from all specialized behavioral and mental health programs across the US [78,79], allowing us to assess a number of organizational constructs.

Capitalizing on NEPEC administrative data, we were also able to capture size/maturity as program inception date, number of available beds and number of patients served in past-year, and number of full-time providers at various educational levels. Formalization was represented by program adherence to national patient admission, discharge, and readmission procedures, as well as through interview discussion regarding provider clarity around the organizational rules for decision-making and implementing changes. Differentiation or division among units was examined through providers’ descriptions on the structured interview of separations between staff from different backgrounds (e.g., psychology, nursing) as well as how different staff sectors communicated and shared practices (e.g., outpatient and residential).

Although there is no standardized measure of decentralization, we devised our own regarding dispersion of authority in decision making around the innovation. Additionally, there are no uniform instruments on slack resources. NEPEC data were used to capture staff to patient ratio and program capacity (including number of unique patients and number of visits).

For absorptive capacity for new knowledge, we devised items or questions for pre-existing knowledge/skill base, ability to learn and integrate new information, and enablement of knowledge sharing. Pre-existing knowledge/skill base was also included in the survey by identifying training level and tenure in the particular program as well as the organization. This was explored further though the interview when assessing overlapping skills-focused questions (see Adopter characteristics section). Ability to learn and integrate new information was assessed in the interview by asking about the provider’s learning experience and experience of use of the innovation and was felt to be adequately captured by interview questions regarding knowledge-seeking. Enablement of knowledge sharing was included in the survey and directly assessed communication patterns and exchange of knowledge.

Greenhalgh et al.’s construct of receptive context for change was judged to be somewhat similar to organizational readiness to change and organizational culture and climate. There are at least 43 organizational readiness for change measures, many of which have poor psychometric properties [80]. Although we considered a number of instruments [81-83], the one that most influenced the construction of our survey was the widely-used Texas Christian University Organizational Readiness for Change [12]. It has demonstrated good item agreement and strong overall reliability.

Similarly, although several tools exist for assessing culture and climate [84-86], most do not adequately capture Greenhalgh’s constructs, and so we developed new items to measure a number of these constructs. We reviewed the Organizational Social Context survey [87], but most of these items were also not representative of Greenhalgh’s constructs. Similarly, we reviewed the Organizational Culture Profile [88]. Although various items shared some commonality with Greenhalgh’s constructs (e.g., ‘being innovative’), we found most items to be relatively unspecific (e.g., ‘fitting in’).

We reviewed several questionnaires that specifically measured organizational leadership. One psychometrically-sound measure, the Multifactor Leadership Questionnaire [89,90] informed our survey item construction. Leadership items examined support for a new initiative from a variety of levels including general mental health and program leaders. We devised an item in order to capture the presence and use of leadership vision.

More specifically, items from the Texas Christian University Organizational Readiness for Change [12] informed our survey items for managerial relations and risk-taking climate. There are no measures of clear goals and priorities and high-quality data capture. We constructed our own items to represent these constructs.

Similarly, no tools were available to capture system readiness for innovation. Many of these constructs are not easily assessed in simple survey items and were therefore included in the interview. System readiness for innovation includes tension for change, innovation-system fit, power balances (support versus advocacy), assessment of implications, dedicated time and resources (e.g., funding, time), and monitoring and feedback.

We were only able to locate one relevant measure of tension for change [91], a rating system developed through interviews with organizational experts to identify factors that influence health system change. Unfortunately, the authors did not provide the specific items utilized, and thus we captured the tension for change in the interview by asking providers about their existing work climate and the perceived need for new treatments. The constructs of innovation-system fit, power balances, assessment of implications, dedicated time and resources, and monitoring and feedback also did not have standardized measures and thus we devised our own questions.

### Outer context

Outer context constructs include socio-political climate, incentives and mandates, interorganizational norm-setting and networks, and environmental stability. There are no standard tools to assess these domains. There are limited measures of sociopolitical climate [8]. We devised questions for the interview regarding environmental ‘pressure to adopt’ to tap into this construct.

Because there were no identified existing measures for incentives and mandates, secondary data sources were used, such as a review of national mandates in provider handbooks from VA Central Office and discussions with one of the co-authors (JR), who is in charge of one of the national evidence-based roll-outs. Likewise for interorganizational norm setting and networks, the team devised items to assess these constructs because no reliable existing measures were available. Environmental stability was derived from interview questions asking if staffing changes had occurred and perceived reasons for changes (e.g., moves, policy changes). This construct clearly overlaps with inner context (e.g., funding clearly translates into resources that are available within the inner context); however, environmental stability is assumed to be affected by external influences. Thus, our group devised survey items and interview questions and used administrative data to represent outer context constructs. While organizational written policies and procedures are likely accessible to most researchers, changes in budgets and funding may not be, particularly for researchers studying implementation from outside an organization. When possible, this type of information should be sought to support the understanding of outer context.

### Implementation process

Factors proposed to represent the process of implementation include decision-making, hands-on approach by leader, human and dedicated resources, internal communication, external collaboration, reinvention/development, and feedback on progress. Consistent with Greenhalgh et al.’s two-phase approach, we primarily captured the implementation process through the interview.

Decision-making was assessed through questions regarding decentralization described above. Because there are no established measures to assess hands-on approach by leader, or human resources issues and dedicated resources, these were developed by group consensus. For internal communication, we asked a question in the interview about whether a provider sought consultation from someone inside their setting regarding the innovation and its implementation. For external collaboration, we also asked a specific question regarding outside formal consultation. We captured the construct of reinvention/development with an interview question concerning how the two innovations are used and whether they had been modified (e.g., number and format of sessions). Because no formal measure for feedback existed, we utilized interview questions from monitoring feedback to capture both constructs. Even though Greenhalgh et al. outline a separate set of constructs for implementation process, these seem to overlap with the other five constructs.

## Discussion

Greenhalgh et al. [1] developed a largely evidence-based comprehensive model of diffusion, dissemination, and implementation that can assist in guiding implementation research as well as efforts to facilitate implementation. Despite numerous strengths of the model, there had been no explicit recommendations for operational definitions or measurement for most of the six identified constructs. Through a systematic literature review of measures for associated constructs and an iterative process of team consensus, our group has taken a first step at operationalizing, and thus testing this model.

We are presently using a mixed-method approach of measurement using quantitative data through survey and administrative data and qualitative data through semi-structured interviews and other artifacts (e.g., review of policies) to examine the implementation of two evidence-based psychotherapies for PTSD nationally within VA [9]. Information from that study should provide knowledge to assist in the refinement of the measures, such as examination of psychometric properties and identifying changes needed to better operationalize the constructs. It will be essential, of course, to test the Greenhalgh et al. model through the use of our mixed-method approach and resulting survey, interview, and administrative data in additional healthcare organizations and settings and with non-mental health interventions. Given the challenge to operationalize such a saturated model, this work should be considered a first step in the advancement of a testable theory. A contextual approach should be taken to strategically determine which constructs are most applicable to the individual study or evaluation. Also, a more in-depth examination of several constructs may be a needed next step.

### Limitations

Some variables potentially important in the process of implementation are not addressed in the Greenhalgh model. For example, there are several adopter characteristics and social cognition constructs that are not included (e.g., intention for behavior change, self-efficacy, memory) [92-94]. Further, in times of increasing fiscal constraint, it is important to note that the model does not consider cost of the innovation itself or costs associated with its implementation, including investment, supply, and opportunity costs (as opposed to available resources from the inner setting) [7].

Other constructs receive mention in the model but likely warrant further refinement and elaboration. For example, while several constructs are similar to organizational culture and climate, concurrent use of other measurement tools may be warranted e.g., [84-87]. Similarly, the concept of leadership received only minimal attention in the Greenhalgh model, even though mental health researchers [10] have found this construct to be influential in implementation. Because the validity of the transtheoretical stages of change model has been questioned [95], alternatives may be needed to capture this important construct.

Other constructs are complicated by overlap (e.g., cosmopolitan, social networks, and opinion leaders) or are similarly applied to more than one domain. One example is feedback on progress, which is listed under the domain implementation process, but the very similar construct monitoring and feedback is listed under the domain system readiness for innovation. Likewise, social networks are captured under both adopter and communication and influence domains. Our measurement process attempted to streamline questioning (both in the survey and interview) by crafting questions to account for redundancy in constructs (e.g., reinvention).

We also chose not to include every construct and sub-construct in the model because their assessment would be burdensome for providers.^1^ In addition, some of these constructs were viewed as best captured in a larger meta-narrative [8] (e.g., assimilation and linkage), mapping the storyline and the interplay of contextual or contradictory information. Like most measures based on participant responses, our survey and interview may be influenced by intentional false reporting, inattentive responding or memory limitations, or participant fatigue.

It is possible that our search terms may not have identified all the relevant measures. For example, there are several other search terms that may have captured the ‘implementation’ domain, such as uptake, adoption, and knowledge transfer. In addition, searching for the specific construct labels from this model assumes that there is consensus in the research community about the meaning of these terms and that no other terms are ever used to label these constructs.

Of course, operationalizing constructs is only one aspect of making a model testable. It also requires information about construct validity, a clear statement of the proposed relationships between elements in the model that would inform an analysis strategy, and a transparent articulation about the generalizability of the model and which contexts or factors might limit its applicability.

In sum, our work here represents a significant step toward measuring Greenhalgh et al.’ comprehensive and evidence-based model of implementation. This conceptual and measurement development now provides for a more explicit, transparent, and testable theory. Despite limitations, the survey and interview measures as well as our use of administrative data described here can enhance research on implementation by providing investigators with a broad measurement tool that includes, in a single questionnaire and interview, most of the many factors affecting implementation that are included in the Greenhalgh model and other overarching theoretical formulations. One important next step will be to evaluate the psychometrics of this measure across various healthcare contexts and innovations and to examine whether the definitional/measurement boundaries are reliable and valid, and further refine our measure. Empirical grounding of the process of implementation remains a work in progress.

## Endnote

^1^See Figure 1. Terms not included in our operationalized survey by construct and sub-construct: System Antecedents for Innovation: Absorptive capacity for new knowledge: ability to find, interpret, recodify and integrate new knowledge; Linkage: Design stage: Shared meanings and mission, effective knowledge transfer, user involvement in specification, capture of user led innovation; Linkage: Implementation stage: Communication and information, project management support; Assimilation: Complex, nonlinear process, ‘soft periphery’ elements.

## Competing interests

The authors declare they have no competing interests.

## Authors’ contributions

JMC, CO and SD conducted a systematic review of articles for possible inclusion and identification of key measurement constructs captured. JMC, CO, SD, JCC, JR and PS participated in the development and refinement of both the quantitative survey measure and semi-structured interview guide. All authors contributed to the drafting, editing and final approval of the manuscript.

## Supplementary Material

Additional file 1 Full Quantitative Survey. Click here for file

Additional file 2 Full Semi-Structured Interview Guide. Click here for file
