# Supplementary material for: Measurement of a model of implementation for health care: toward a testable theory
Source: Implement Sci. 2012 Jul 3;7:59. doi: 10.1186/1748-5908-7-59 (PMC3541168; doi:10.1186/1748-5908-7-59)
Supplement: Additional file 1 — Full Quantitative Survey. [file 1748-5908-7-59-S1.doc]

| **Construct** | **Survey Question** |
| --- | --- |
| **Innovation** | |
| Relative advantage | [The treatment] is more effective than other therapies I have used.  [The treatment] is more convenient than other therapies I have used. |
| Compatibility | Using [the treatment] fits well with the way I like to work.  [The treatment] is aligned with my clinical judgment. |
| Complexity | [The treatment] is clear and understandable.    [The treatment] is easy to use. |
| Trialability | [The treatment] can be tested out with patients without disrupting their overall therapy.  It is easy to try out [the treatment] and see how it performs. |
| Observability | It is easy to tell whether patients are benefitting from [the treatment].  [The treatment] produces improvements in my patients that I can actually see. |
| Potential for reinvention | [The treatment] can be adapted to fit my treatment setting.  [The treatment] can be adapted to meet the needs of my patients. |
| Risk | Using [the treatment] includes a risk of worsening my patients’ symptoms.  Using [the treatment] includes a risk of doing more harm than good. |
| Task issues | Using [the treatment] improves the quality of work that I do.  Using [the treatment] makes it easier to do my job. |
| Nature of knowledge | The knowledge required to learn [the treatment] can be effectively taught.  The skills required to implement [the treatment] can be effectively taught. |
| Technical support | [The treatment] manual is helpful.  [The treatment] has helpful supportive materials for patients. |
| **Adopter Characteristics** | |
| Needs | I feel the need to learn additional therapies to help my patients with their symptoms. |
| Motivation | I do not think I need to change my therapy techniques.  I think my therapy techniques could use some improvement.  I am actively working on improving my therapy techniques.  I have been successful in improving my therapy techniques, but ongoing professional support would help to maintain my efforts. |
| Values and goals | I prefer training content based on scientific evidence.  I think it is important that providers use evidence-based treatments. |
| Skills | I have not attended any training in [the treatment].  I read [the treatment] manual on my own.  I have participated in informal training in [the treatment].  I attended the [x] day workshop in [the treatment].  I completed case consultation [in the treatment].  I achieved certification in [the treatment]. |
| Learning style | I learn effectively by reading.  I learn effectively through experience, such as role play or work with actual patients.  I learn best through trial and error in applying a treatment with my patients. |
| Locus of control (internal and external) | My life is determined by my own actions. (internal)  I have found that what is going to happen will happen. (external) |
| Tolerance of ambiguity | I am comfortable not being able to predict how a new treatment will work for a particular patient. |
| Tenure | How many years have you been practicing clinically?  How long have you been working in your treatment setting?  What year did you receive your highest degree? |
| Knowledge-seeking | I try to regularly improve my psychotherapy skills.  I try to learn as much as I can about new approaches to psychotherapy.  I feel the need to learn additional [treatments] with my patients. |
| Cosmopolitan | I attend national conferences related to my work with patients. |
| **Communication and Influence** | |
| Social networks | I have colleagues in my treatment setting who are sources of advice and information in my clinical work.  I have colleagues in my treatment setting who are sources of peer support in my clinical work.  When you need information or advice about [treatment] to which other providers in your treatment setting do you usually turn? |
| Homophily | N/A |
| Peer opinion (leader) | I have at least one colleague in my research setting whom I trust as a resource for information regarding [the treatment]. |
| Marketing | N/A |
| Expert opinion (leader) | I am a consultant or trainer in an evidence-based psychotherapy. |
| Champions | N/A |
| Boundary spanner | I have at least one readily accessible person that connects me with [treatment] experts. |
| Change agent | N/A |
| **System Antecedents for Innovations** | |
| Size/Maturity | N/A |
| Formalization | N/A |
| Differentation | N/A |
| Decentralization | N/A |
| Slack resources | N/A |
| **Absorptive Capacity for Knowledge** | |
| Enablement of knowledge sharing | Staff at my treatment setting actively share information and knowledge.  There are adequate communication systems to support information exchange in my treatment setting. |
| Pre-existing knowledge/skill base | What is your most advanced degree?  Please list your primary profession. |
| Ability to learn and integrate new information | N/A |
| Leadership and vision | Program leadership in my treatment setting support new initiatives.  Program leaders in my treatment setting have articulated a vision and mission that we advance as a program.  Mental health leadership at my facility support new initiatives.  Executive leadership at my facility supports new initiatives. |
| Managerial relations | Program leaders and staff in my treatment setting have good working relationships.  Leaders and staff work together effectively in my treatment setting. |
| Risk-taking climate | My work environment encourages experimentation with new practices.  Leaders in my treatment setting reward risk-taking to improve patient care. |
| Clear goals and priorities | I have a clear understanding of my work environment.  The goals and priorities of my treatment setting are clear and consistent. |
| High quality data capture | Outcome data are routinely used in my treatment setting to monitor treatment.  Outcome data are routinely used in my treatment setting for quality improvement. |
| **System Readiness for Innovation** | |
| Tension for change | N/A |
| Innovation-system fit | N/A |
| Power balances | N/A |
| Assessment of implications | N/A |
| Dedicated time and resources | There is adequate time to implement [the treatment] in my treatment setting.  There is adequate consultation to support me in implementing [the treatment] in my treatment setting. |
| Monitoring feedback | N/A |
| **Outer Context** | |
| Socio-political climate | N/A |
| Incentives and mandates | Mental health leaders at my facility actively encourage me to use [the treatment].  I am expected to use [the treatment] as part of my job. |
| Inter-organizational norm setting and networks | N/A |
| Environmental stability | N/A |
| **Implementation Process** | |
| Decision-making | N/A |
| Hands on approach by leaders | Program leaders in my treatment setting are actively involved in supporting [the treatment] initiatives.  Program leaders in my treatment setting are actively involved in daily program activities. |
| Human resources issues | N/A |
| Internal communication | N/A |
| External collaboration | N/A |
| Reinvention | N/A |
| Feedback | N/A |

*Item response options are available from the first author.
