# Supplementary material for: Measurement of a model of implementation for health care: toward a testable theory
Source: Implement Sci. 2012 Jul 3;7:59. doi: 10.1186/1748-5908-7-59 (PMC3541168; doi:10.1186/1748-5908-7-59)
Supplement: Additional file 2 — Full Semi-Structured Interview Guide. [file 1748-5908-7-59-S2.doc]

**SEMI-STRUCTURED INTERVIEW**

Thank you so much for agreeing to the interview. We are interested in finding out treatment providers’ awareness of and experience with______. There are no right or wrong answers.

What is your professional background?

What is your theoretical orientation?

What is your preferred way of learning a new treatment? (Do you prefer using pictures, images, and spatial understanding; using sound and music; using written words; using your body, hands and sense of touch; using logic and reasoning; learning in groups or with other people; working **alone and using self-study?)**

What was your interest in attendance and involvement with training in [the treatment]?

Have you been trained in [the treatment]?

If so, how did you get involved in the trainings (e.g., volunteered, told you had to go)?

How far in the training process did you go (e.g., attended multiple day workshop, completed case consultation, achieved certification, became a trainer)?

What was the experience of learning [the treatment] like? Were there elements that were more or less difficult to learn? How did it fit with your previous beliefs and practices?

What was your experience like bringing [the treatment] into your practice?

How were you persuaded [the treatment] would meet your clinical needs and those of your patients?

What proportion of patients are using [the treatment] with?

How do you determine when a patient is appropriate and/or ready for [the treatment]? How effective is [the treatment] when presenting problems are more acute, severe or complicated?

How do you (or your program) use [the treatment)? Do you use the full protocol (exact number of sessions, in order, including all content), or have the protocols required modification?

To what extent does [the treatment] fit with interventions offered at your treatment setting?

Have you had to make any changes to your program structure to accommodate the use of [the treatment]?

Have there been any unintended benefits or consequences to implementing [the treatment]?

How do different levels of care communicate and share [the treatment] (e.g., residential and outpatient care)?

How are you able to implement the treatment in regards to available time and resources?

What are some of the supports or structures that are helpful in implementing [the treatment]?

Did you seek consultation from someone in your setting regarding [the treatment] or its implementation?

Do you have access to and have you sought consultation from an official expert?

Did you seek consultation from someone outside of your setting regarding [the treatment] or its implementation?

How did the program make the decision to implement [the treatment] (or not)?

Did other providers in your setting see a need to make changes to the program and [treatment] approaches?

Did you feel pressure to adopt [the treatment]?

To what extent is [the treatment] supported by program leaders and supervisors?

Was there an individual(s) responsible for facilitating implementation of [the treatment]?

Were there opportunities for you to provide and receive feedback about the implementation process?

Were there key individuals in your program that rallied to support and promote [the treatment]?

What staffing or funding changes occurred in the recent past?

Do program leaders and staff work well together?

Was their agreement among providers, directors and management regarding implementation?

Do you feel that the rules are clear in your organization for making decisions and implementing changes?

What is your understanding of expectations in regards to [treatment] implementation and the associated rewards and penalties?

Does your work environment allow opportunities to experiment with new treatments?
